# Degree of tumour vascularity correlates with drug accumulation and tumour response upon TNF-*α*-based isolated hepatic perfusion

**DOI:** 10.1038/sj.bjc.6600707

**Published:** 2003-01-28

**Authors:** B van Etten, M R de Vries, M G A van IJken, T E Lans, G Guetens, G Ambagtsheer, S T van Tiel, G de Boeck, E A de Bruijn, A M M Eggermont, T L M ten Hagen

**Affiliations:** 1 Department of Surgical Oncology, University Hospital Rotterdam-Daniel den Hoed Cancer Center, Rotterdam, The Netherlands; 2 Laboratory for Organic Analytical Chemistry, University of Antwerp, Belgium; 3 Laboratory for Experimental Oncology, University of Leuven, Belgium

**Keywords:** liver metastases, isolated hepatic perfusion, TNF-*α*, melphalan, microvessel density

## Abstract

Isolated hepatic perfusion (IHP) with melphalan with or without tumour necrosis factor alpha (TNF-*α*) is currently performed in clinical trials in patients with hepatic metastases. Previous studies led to the hypothesis that the use of TNF-*α* in isolated limb perfusion causes specific destruction of tumour endothelial cells and thereby induces an increased permeability of tumour vasculature. However, whether TNF-*α* contributes to the therapeutic efficacy in IHP still remains unclear. In an *in vivo* rat liver metastases model we studied three different tumours: colon carcinoma CC531, ROS-1 osteosarcoma and BN-175 soft-tissue sarcoma which exhibit different degrees of vascularisation. IHP was performed with melphalan with or without the addition of TNF-*α*. IHP with melphalan alone resulted, in all tumour types, in a decreased growth rate. However in the BN-175 tumour addition of TNF-*α* resulted in a strong synergistic effect. In the majority of the BN-175 tumour-bearing rats, a complete response was achieved. *In vitro* cytoxicity studies showed no sensitivity (CC531 and BN-175) or only minor sensitivity (ROS-1) to TNF-*α*, ruling out a direct interaction of TNF-*α* with tumour cells. The response rate in BN-175 tumour-bearing rats when TNF-*α* was coadministrated with melphalan was strongly correlated with drug accumulation in tumour tissue, as only in these rats a five-fold increased melphalan concentration was observed. Secondly, immunohistochemical analysis of microvascular density (MVD) of the tumour showed a significantly higher MVD for BN-175 tumour compared to CC531 and ROS-1. These results indicate a direct relation between vascularity of the tumour and TNF-*α* mediated effects. Assessment of the tumour vasculature of liver metastases would be a way of establishing an indication for the utility of TNF-*α* in this setting.

Tumour necrosis factor alpha (TNF-*α*) is a cytokine with an interesting potential in the treatment of cancer (ten Hagen *et al*, 2001). When administered systemically it is accompanied with severe toxicity; however, especially when TNF-*α* in combination with chemotherapy is used locoregionally without systemic exposure, it has very potent antitumour effects. Clinical trials of isolated limb perfusion (ILP) with recombinant human TNF-*α* and melphalan resulted in high complete response rates of 75–90% in patients with in-transit melanoma and unresectable sarcoma of the extremities (Lienard *et al*, 1992; Eggermont *et al*, 1996a,1996b). This is in contrast to ILP with melphalan alone, which is relatively effective against small in-transit melanoma metastases (Lejeune *et al*, 1989), but achieves very poor results against large tumours such as soft-tissue sarcomas (McBride, 1974; Hoekstra *et al*, 1987; Thompson and Gianoutsos, 1992).

In order to elucidate the mechanism of TNF-*α*, several studies have been performed. In our preclinical ILP model, we observed drastic alterations in tumour microvasculature integrity (Nooijen *et al*, 1996). Rüegg *et al*, (1998) demonstrated elegantly that TNF-*α* in combination with IFN-*γ* induced functional downregulation of *α*v*β*3, resulting in detachment of the endothelial cells of the tumour vasculature. Moreover, angiographic studies performed in patients pre- and post-TNF-*α* perfusion showed selective destruction of tumour-associated vasculature and histologic studies demonstrated haemorrhagic necrosis of the tumour (Olieman *et al*, 1997). Recently, we demonstrated, what we consider a key explanation for the potent synergy between TNF-*α* and chemotherapy, an up to six-fold increased intratumoural melphalan or doxorubicin concentration in rat sarcomas after ILP when high-dose TNF-*α* was coadministrated (de Wilt *et al*, 2000; van der Veen *et al*, 2000). These findings led to the hypothesis that TNF-*α* causes specific destruction of tumour endothelial cells and thereby induces an increased permeability of tumour vasculature.

As a result of the favourable experience with the ILP system, other isolated perfusion settings have been developed (van der Veen *et al*, 1999; van Ijken *et al*, 2000). Especially, the liver offers superb opportunities for isolated perfusion. Irresectable liver metastases are a significant clinical problem. Isolated hepatic perfusion (IHP) with melphalan with or without TNF-*α* is technically feasible and is currently performed in clinical trials in patients with hepatic metastases (Alexander *et al*, 1998a,1998b; Vahrmeijer *et al*, 2000). Whether TNF-*α* contributes to the therapeutic efficacy in IHP still remains unclear.

Based on our findings in the ILP studies, it is indicated to study whether TNF-*α* can improve tumour response in different tumours after IHP and, if so, to investigate the capability of TNF-*α* to augment drug accumulation in this perfusion setting. By addressing this issue, the usefulness of TNF-*α* in IHP might become clear. Since the tumour-associated vasculature is the target of TNF-*α*, we expect that tumour microvessel density (MVD) is a predictor of the potentiating effect of TNF-*α* during isolated perfusions. Here we present data that indicate that the antitumour effect of TNF-*α* is correlated with the tumour microvessel density.

## MATERIALS AND METHODS

### Rat liver metastases model

We used male inbred WAG/RIJ or Brown-Norway (BN) strain rats, weighing 250–300 g, obtained from Harlan-CPB (Austerlitz, The Netherlands). The rats were fed a standard laboratory diet. All animals were housed under standard conditions of light and accommodation. The protocol was approved by the committee for animal research of the Erasmus University, Rotterdam, The Netherlands. The experimental protocols adhered to the rules outlined in the Dutch Animal Experimentation Act of 1977 and the published Guidelines of the UKCCCR for the Welfare of Animals in Experimental Neoplasia (UKCCCR, 1998).

Three different tumours were used in this study. The weakly immunogenic colon carcinoma CC531 is a 1,2-dimethylhydrazine-induced, moderately differentiated adenocarcinoma transplantable in syngeneic WAG/RIJ rats. The estimated doubling *in vivo* is about 6–8 days. The spontaneously originated nonimmunogenic osteosarcoma ROS-1 is also transplantable in the WAG-RIJ rat and in the liver metastases model it has a mean doubling time of about 4–5 days. The spontaneously originated nonimmunogenic soft-tissue sarcoma BN-175 is the fastest growing tumour of the tumours tested, with an estimated doubling time *in vivo* of about 2–3 days and is transplantable in syngeneic BN rats. Following a standardised protocol, small viable tumour fragments of CC531, ROS-1 or BN-175 tumour fragments of 1×2 mm^2^ were implanted under the liver capsule, one on the left and one on the right side of the left liver lobe, using a 19 G Luerlock needle. Experiments started at a fixed tumour diameter between 5 and 6 mm. When tumours reached a size of 20 mm in diameter or animals showed obvious signs of discomfort the animals were killed.

### Drugs

Recombinant human TNF-*α* (4.9–5.8×10^7^ U mg^−1^) was provided as a kind gift by Boehringer Ingelheim GmbH, Ingelheim/Rhein, Germany. Melphalan (L-pam, Alkeran, Wellcome Ltd, London, UK) was obtained as a sterile powder (100 mg) that was dissolved aseptically using solvent and diluent provided by Burroughs Wellcome (London, UK).

### Isolated hepatic perfusion

**Figure 1:**
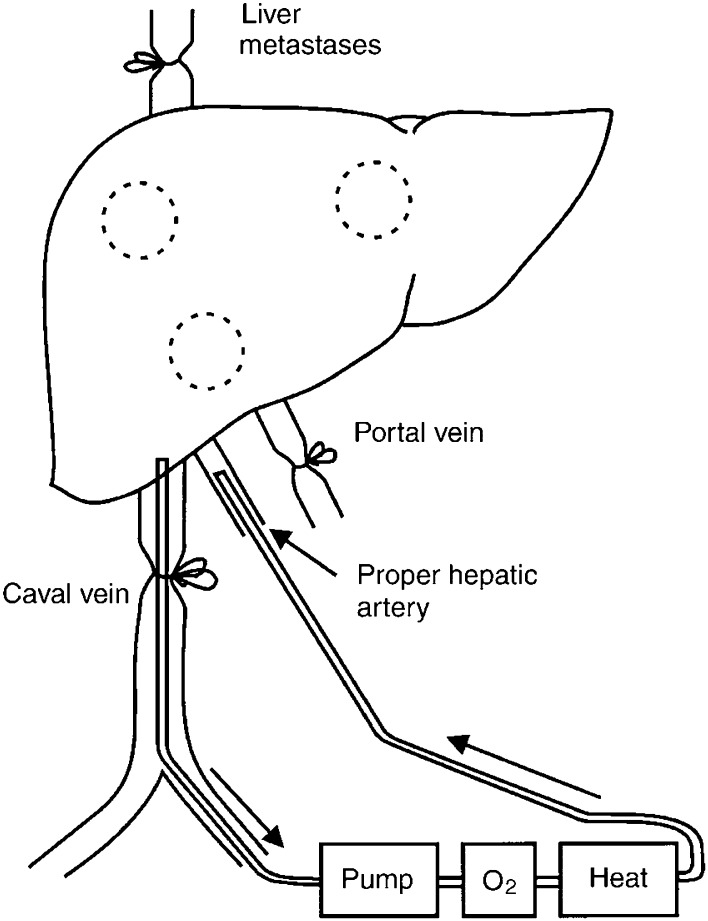
Schematic representation of an IHP.

This rat isolated liver perfusion model has been described in detail earlier by van IJken *et al*, (2000). A schematic representation is shown in Figure 1

. Anaesthesia was induced and maintained with ether (Merck, Darmstadt, Germany). During the surgical procedure, with an average duration of 60–75 min, rats were kept at a constant temperature using a warmed mattress. A mid-line laparotomy was performed and the hepatic ligament exposed. The gastroduodenal side branch of the common hepatic artery was cannulated, positioning the tips of the cannula (0.025 outer diameter (OD), 0.012 in inner diameter (ID) (Dow Corning, MI, USA)) in the proper hepatic artery. Through a small inguinal incision the femoral vein was exposed. To collect hepatic venous outflow a silicon cannula (0.047 OD, 0.025 in ID) (Dow Corning, MI, USA) was introduced in the femoral vein and moved up into the caval vein positioning the tip of the cannula at the level of the hepatic veins.

Isolation of the hepatic vascular bed was obtained by temporarily ligating the common hepatic artery and the portal vein. The venous outflow limb was isolated by temporarily clamping the supra-hepatic caval vein and by applying a temporary ligature around the infra-hepatic caval vein containing the cannule, cranial to the right adrenal vein. The mesenteric artery was temporarily clamped in order to reduce splanchnic blood pressure. The circuit was primed with 10 ml Haemaccel (Behring Pharma, Amsterdam, The Netherlands). Arterial flow of 5 ml min^−1^ was maintained with a low-flow roller pump (Watson Marlow type 505 U, Falmouth, UK). Rats were perfused for ten min with oxygenated Haemaccel in which melphalan and/or TNF-*α* was dissolved. This short perfusion time was used as we observed rapid clearance of melphalan from the perfusate in this time frame. Secondly, perfusion of the liver beyond 10 min may increase the risk for tissue damage to the liver, but also to the gut as blood flow to the gut is impaired during the perfusion. Afterwards a washout was performed by perfusing with 10 ml of oxygenated Haemaccel. Heparin (50 IU) (Heparine Leo, The Netherlands) was added to the perfusate. The perfusate was oxygenated in a reservoir with a mixture of O_2_/CO_2_ (95% : 5%) and was kept at 38–39°C by means of a heat exchanger and a warm water bath. A temperature probe was positioned in the lumen of the arterial catheter, 5 cm from the catheter tip.

Following the washout procedure, the clamps on caval vein, portal vein, hepatic artery and mesenteric artery were released. The gastroduodenal artery and femoral vein were ligated and the gastroduodenal and femoral cannulas were removed.

### *In vivo* antitumour efficacy study

Treatment started at a fixed tumour size of 5–6 mm in diameter. Rats were perfused in random order. In a pilot dose finding study performed for each tumour type the melphalan dose inflicting a *partial tumour response* was chosen for this study. So in the case of additive or synergistic effect of TNF-*α* on melphalan this could still be demonstrated in the growth curves of the tumours. All animals underwent IHP only once. CC531-bearing rats were treated with 50 *μ*g melphalan (*n*=6), 20 *μ*g TNF-*α* (*n*=6) or a combination of 50 *μ*g melphalan and 20 *μ*g TNF-*α* (*n*=6). ROS-1-bearing rats were perfused with 50 *μ*g melphalan (*n*=6), 20 *μ*g TNF-*α* (*n*=8), or a combination of 50 *μ*g melphalan and 20 *μ*g TNF-*α* (*n*=6). In the BN-175-bearing rats perfusions were carried out with 200 *μ*g melphalan (*n*=6), 20 *μ*g TNF-*α* (*n*=6), or a combination of 200 *μ*g melphalan and 20 *μ*g TNF-*α* (*n*=6). After IHP tumour size was measured via a small midline laparotomy every fourth day. Tumour volume was calculated by using the following formula: tumour volume=A^2^×B×0.4, where *B* is the largest diameter and *A* the diameter perpendicular to B, measured with a standardised calliper. In every treatment group, sham perfused rats (*n*=6) and untreated control rats (*n*=5) were included.

### *In vitro* cytotoxicity assay

CC531 and BN-175 cells were grown in RPMI 1640 and ROS-1 cells in modified Eagle's medium (Gibco BRL, Paisley, UK) supplemented with 10% foetal calf serum (Harlan/Sera-Lab, UK), 1% penicillin (5000 IU ml^−1^), 1% streptomycin (5000 IU ml^−1^) and 1% L-glutamine (200 mM) (all Gibco BRL) in a humidified incubator at 37°C and 5% CO_2_. Before usage, the cells were trypsinised (1 min, 37°C), centrifuged (5 min, 700 g), resuspended and the viability measured by trypan blue exclusion. For *in vitro* testing of proliferation inhibition, 1.0×10^4^ viable cells were seeded in flat-bottomed 96-well microtiter plates (Costar, USA). After 24 h the cells were incubated with different concentrations of TNF-*α* for 72 h ranging from 0 to 10 *μ*g ml^−1^. Afterwards, cells were washed with PBS and fixed for 1 h with 10% trichloroacetic acid at 4°C. Growth of tumour cells was measured using the sulpharhodamine-B assay according to the method of Skehan *et al*, (1990). Tumour cell proliferation was measured using the formula: tumour growth=(test well/control)×100%. Five independent tests were performed for each point on the line.

### Measurement of melphalan in tissue

After 5 min of the restoration of the circulation, the perfused tumour and part of the liver were excised. The tissues were immediately frozen in liquid nitrogen to stop metabolism of melphalan and stored at –80°C. Tumour and liver tissues were homogenised in 2 ml acetonitrile (Pro 200 homogenizer, Pro Scientific, CT, USA) and centrifuged at 2500 **g**. Melphalan was measured in the supernatant by gas chromatography–mass spectrometry (GC–MS). *p*-[Bis(2-chloroethyl)amino]-phenylacetic acid methyl ester was used as an internal standard. Samples were extracted over trifunctional C18 silica columns. After elution with methanol and evaporation, the compounds were derivatised with trifluoroacetic anhydride and diazomethane in ether. The stable derivates were separated on a methyl phenyl siloxane GC capillary column and measured selectively by single-ion monitoring GC–MS in the positive EI mode described earlier by Tjaden and Bruijn (1990).

### Assessment of tumour microvessel density by immunohistochemistry

Cryosections of tumours were fixed for 15 min with 4% formaldehyde. After rinsing with PBS, sections were incubated for 1 h with 1 : 10 PBS diluted, mouse-anti-rat-endothelial cell antibody (RECA-1, Instruchemie, Hilversum, The Netherlands). For the negative control an aspecific mouse IgG was used (Santa Cruz Biotechnology, Santa Cruz, CA, USA). Thereafter, sections were rinsed with PBS and incubated for 1 h with 1 : 100 diluted, in 5% normal rat serum in PBS, goat-anti-mouse peroxidase-labelled antibody (DAKO, Carpinteria, CA, USA). After rinsing with PBS, positive cells were revealed by immunoperoxidase reaction with DAB solution (DAB-kit, DAKO) and counterstained with haematoxylin. For microvessel quantification two independent persons performed a blinded analysis. Positive cells were counted in three different high-power fields (magnification ×160) in each slide according to the method of Bosari *et al*, (1992). In total, three slides per tumour and three tumours per tumour type were evaluated.

### Statistical analysis

*In vitro* bioassays and *in vivo* tumour response results were evaluated for statistical significance with the Mann–Whitney *U*-tests with SPSS8.0 for Windows 98. Mann–Whitney *U*-test was used to compare melphalan concentrations in different groups and Kruskal–Wallis test to compare number of positive cells in different tumours. A significance level of *P*<0.05 was used in all analyses.

## RESULTS

### Tumour response after isolated hepatic perfusion

**Figure 2:**
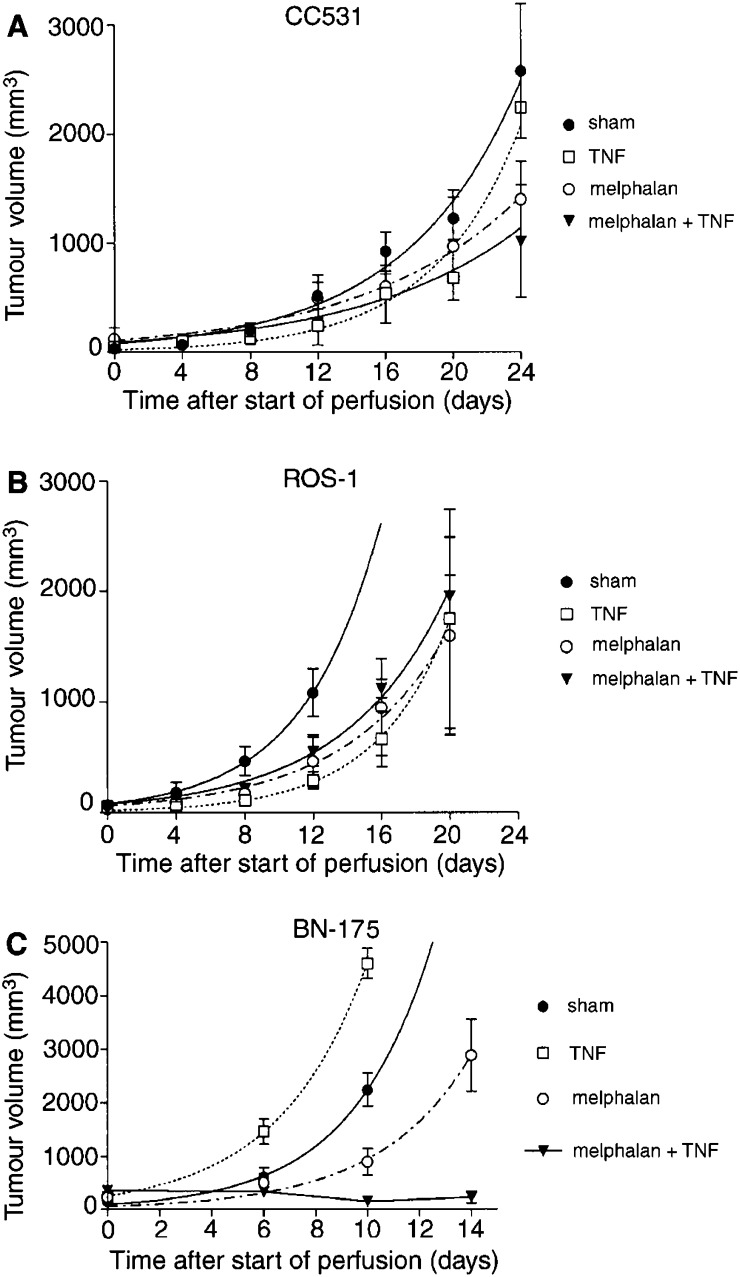
Growth curves of *in vivo* tumours after IHP. Each group contained at least six animals. Mean values (±s.e.m.) are shown; (**A**) CC531, (**B**) ROS-1, (**C**) BN-175.

The antitumour efficacy of IHP with melphalan with or without TNF-*α* was evaluated for the CC531, ROS-1 and BN-175 tumour starting at an equal size of 5–6 mm in diameter. In all groups, sham IHPs with only perfusion medium were performed. The graphs in Figure 2

show the growth curves of CC531 tumour (a), ROS-1 (b) and BN-175 (c) after IHP with melphalan, TNF-α, both or after sham perfused rats. Perfusion with melphalan alone significantly reduced tumour growth rates compared with sham perfused animals in all tumour types. When IHP was performed in BN-175-bearing rats with the combination of melphalan and TNF-*α*, a dramatically enhanced tumour response was observed in all animals. This is a significant reduction of mean tumour volume compared with rats perfused with either TNF-*α* only or melphalan alone (*P*<0.005 and <0.01, respectively). In the CC531 or ROS-1 tumours, synergy between TNF-*α* and melphalan was not observed.

### *In vitro* cytotoxicity assay

**Figure 3:**
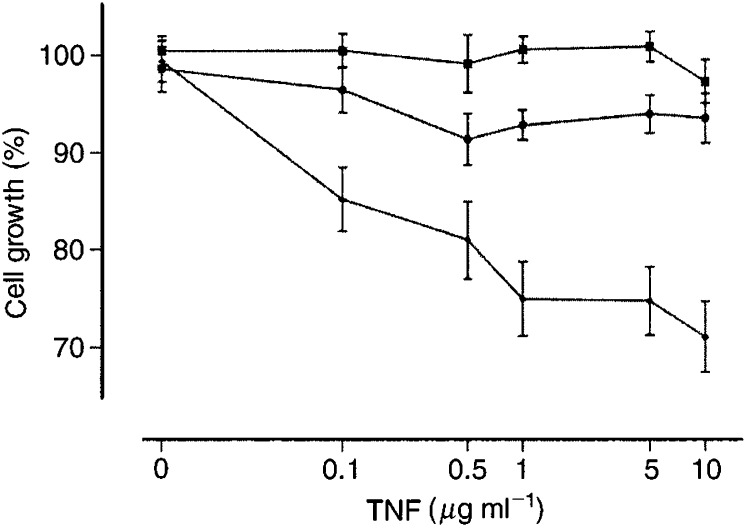
*In vitro* growth curves of tumour cells upon exposure to TNF-*α*; CC531 (•), ROS-1 (⧫), BN-175 (▪). Six independent assays were performed in duplicate for each point on the line. Mean values (±s.e.m.) are shown.

The effect of TNF-*α* on the growth of tumour cells *in vitro* was determined to evaluate whether the synergistic effect of TNF-*α* could be related to direct tumour cell toxicity. The calculated concentration of TNF-*α* in the perfusate during IHP *in vivo* is about 1.5 *μ*g ml^−1^. So *in vitro* tumour cells were exposed to a range of TNF-*α* concentrations varying from 0 to 10 *μ*g ml^−1^. The growth curves are shown in Figure 3

. It is demonstrated that the BN-175 and the CC531 tumour cell line did not show significant sensitivity to TNF-α. Only the ROS-1 tumour cells were moderately sensitive to TNF-*α*, a growth inhibition of up to 30% at 10 *μ*g ml^−1^ was observed.

### Melphalan concentration in tumour and liver tissue

**Figure 4:**
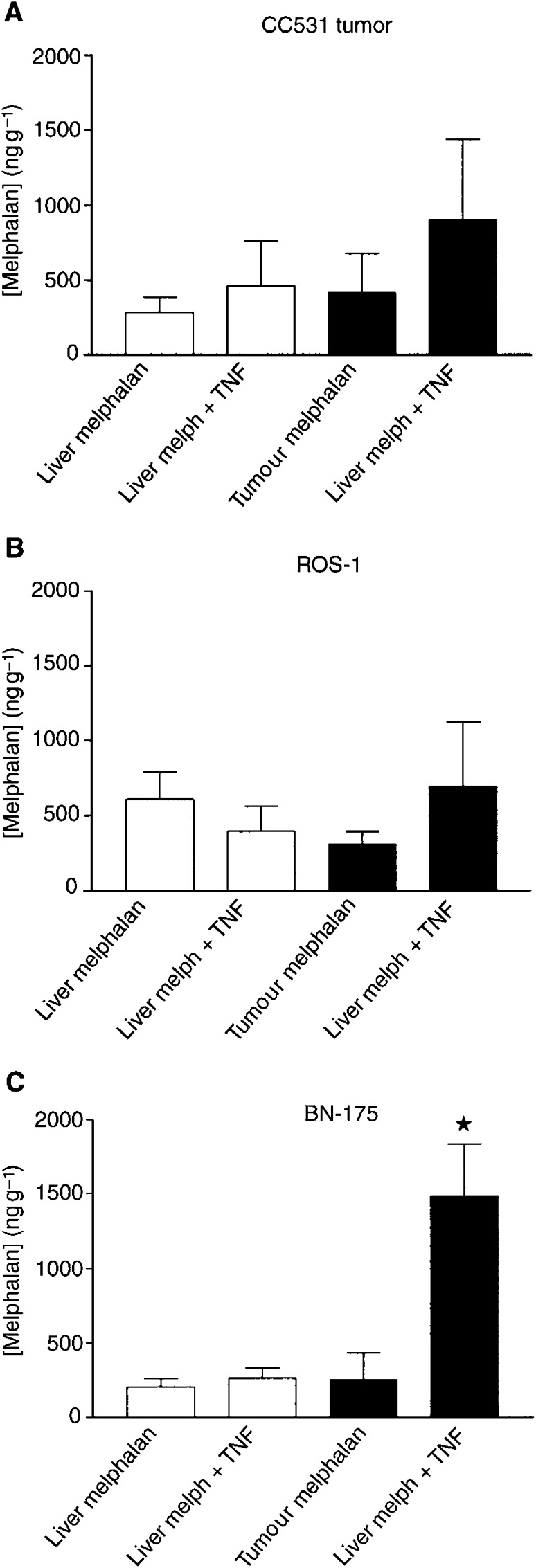
Melphalan concentrations in liver and tumour tissue after IHP with melphalan with or without TNF-α. Six IHPs were performed per tumour type. Mean values (±s.d.) are shown. (^*^=*P*<0.05 *vs* tumour melphalan concentration after IHP with melphalan alone); (**A**) CC531, (**B**) ROS-1, (**C**) BN-175.

In this perfusion setting, in which the dose of TNF-*α* is 20% of the dose used in ILP, an enhanced drug accumulation in tumour tissue might take place as well, as observed after TNF-*α* based ILP. In order to investigate this mechanism, melphalan concentrations were measured in tumour and liver tissues after IHP with melphalan with and without TNF-*α*. In the CC531 and ROS-1, tumours, melphalan concentration did not increase significantly after IHP with melphalan and TNF-*α* (Figure 4A,B

). After IHP with melphalan alone in the BN175 tumour-bearing rats the melphalan concentration in tumour and liver tissue was equal (Figure 4C). After IHP with TNF-*α* however a more than 5-fold increase of melphalan in tumour tissue is measured compared to tumour tissue after IHP without TNF-*α*; (*P*<0.05). So an augmented drug accumulation can also be achieved in the IHP setting when TNF-*α* is coadministered.

### Assessment of tumour microvessel density

**Figure 5:**
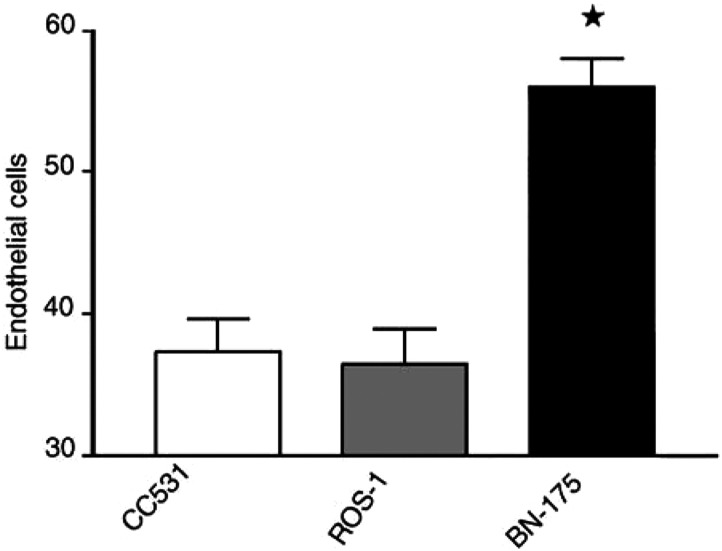
Microvessel count of CC531, ROS-1 and BN-175 tumours. Mean values (±s.e.m.) are shown (^*^=*P*<0.001 *vs* CC531 and *vs* ROS-1).

We already hypothesised that TNF-*α* by increasing leakage of tumour vessels enhances intratumoural concentrations of chemotherapeutics. The increased uptake of melphalan might therefore be correlated with the microvessel density (MVD) of the tumour. Quantification of the MVD was performed by immunohistochemical staining of endothelial cells. The microvessel count of the colon carcinoma CC531 and the osteosarcoma ROS-1 was equal (Figure 5

). The soft-tissue sarcoma BN-175 however showed a significantly higher MVD than CC531 and ROS-1. These results indicate a relations between vascularity of the tumour and TNF-*α*-mediated effects.

## DISCUSSION

In the present study, we demonstrated that addition of TNF-*α* to IHP with melphalan results in strongly improved response rates in a tumour with high vascular density. *In vitro*, no or only minor sensitivity of tumour cells to TNF-*α* was found. Even in ROS-1 tumours, which are moderately sensitive to TNF-*α* *in vitro*, IHP with TNF-*α* alone showed no tumour response. These data indicate strongly that *in vivo* indirect mechanisms mediated by TNF-*α* in combination with melphalan determine antitumour effects in IHP. Our data support the notion that this indirect mechanism is the selective destructive effect of TNF-*α* on the tumour-associated vessels, thereby increasing vascular permeability (Nooijen *et al*, 1996; Ruegg *et al*, 1998). To investigate this hypothesis, the melphalan uptake in liver and tumour tissue was measured after IHP with or without TNF-*α*. Tumour melphalan concentrations were increased in all tumours but varied significantly in a tumour-type-dependent way. Moreover, enhanced uptake of melphalan by healthy liver was not observed. With TNF-*α* alone, at the most some tumour growth was observed. Only the combination of TNF-*α* and melphalan resulted in a complete tumour response in the BN175 tumour. To elucidate this tumour-type-dependent response, the MVD of the tumours was determined. We expected a higher tumour vascularity in this tumour. Indeed a significantly higher MVD compared to the CC531 and ROS-1 tumours could be demonstrated. This indicates that TNF-*α* has specific tumour vascular mediating capacity in this perfusion model, which results in enhanced tumour responses in highly vascularised tumours. As a result of our findings in ILP and now also in IHP, we know that TNF-*α* is able to augment the accumulation of melphalan. The presence or lack of TNF-*α*-mediated synergy appeared to be independent of tumour size as also in smaller (diameter 3–4 mm) or bigger (7–8 mm) tumours comparable tumour responses were observed (data not shown). We are of the opinion that this observation is essential in understanding and explaining the impressive responses demonstrated.

Changes in vascular permeability in patients who underwent IHP with TNF-*α* was studied by Alexander *et al*, (1998). Vascular permeability was measured by diffusion of radiolabelled ^131^I albumin in liver and tumour tissue. A significant increase of the ^131^I albumin postperfusion could be demonstrated compared to levels ^131^I albumin measured before perfusion. However, this rise was equal in tumours perfused with or without TNF-*α*. A TNF-*α* independent mechanism of the increased endothelial permeability was suggested by the authors. However, in the present study, we demonstrated that TNF-*α* is effective in increasing vascular permeability for melphalan selectively in tumour tissue. A more important finding, however, is that this effect could only be found in the highly vascularised BN-175 tumour. The results of Alexander *et al*, reported on intratumoural ^131^I albumin concentrations were mainly based on colorectal carcinoma liver metastases. In hypovascular rat colon carcinoma, we also could not find an increase of melphalan intratumourly. We therefore hypothesize that the usual hypovascularity of colorectal metastases in patients explains the lack of TNF-benefit in the experience as described by Alexander in patients, which correlates closely to our observations in our hypovascular colon cancer liver metastases model in rats.

IHP with melphalan and TNF-*α* performed in patients with metastases of ocular melanoma or leiomyosarcoma showed overall response rates of 50–52% (Lindner *et al*, 1999; Alexander *et al*, 2000). Both tumour types are highly vascularised. A prolonged duration of response was found in melanoma patients: 14 months after IHP with TNF-*α* *vs* 6 months after IHP without TNF-*α* (Alexander *et al*, 2000). After IHP with melphalan with or without TNF-*α* in patients with colorectal liver metastases the mean duration of response was in both groups 8–10 months (Alexander *et al*, 1998a,1998b; Bartlett *et al*, 2001). The data we now present and the first reports of IHP in melanoma and sarcoma liver metastases strongly indicate that in these patients TNF-*α* has therapeutic potential in IHP. In patients with colorectal liver metastases however, IHP with melphalan alone may well be just as effective as combined with TNF-*α*. Assessment of the degree of tumour vasculature of liver metastases would be a way of establishing an indication for the utility of TNF-*α* in this setting.

## References

[bib1] Alexander HR, Brown CK, Bartlett DL, Libutti SK, Figg WD, Raje S, Turner E (1998a) Augmented capillary leak during isolated hepatic perfusion (IHP) occurs via tumour necrosis factor-independent mechanisms. Clin Cancer Res 4: 2357–2362. 9796965

[bib2] Alexander HR, Libutti SK, Bartlett DL, Puhlmann M, Fraker DL, Bachenheimer LC (2000) A phase I-II study of isolated hepatic perfusion using melphalan with or without tumour necrosis factor for patients with ocular melanoma metastatic to liver. Clin Cancer Res 6: 3062–3070. 10955785

[bib3] Alexander HRJ, Bartlett DL, Libutti SK, Fraker DL, Moser T, Rosenberg SA (1998b) Isolated hepatic perfusion with tumour necrosis factor and melphalan for unresectable cancers confined to the liver. J Clin Oncol 16: 1479–1489. 9552055 10.1200/JCO.1998.16.4.1479

[bib4] Bartlett DL, Libutti SK, Figg WD, Fraker DL, Alexander HR (2001) Isolated hepatic perfusion for unresectable hepatic metastases from colorectal cancer. Surgery 129: 176–187. 11174711 10.1067/msy.2001.110365

[bib5] Bosari S, Lee AK, DeLellis RA, Wiley BD, Heatley GJ, Silverman ML (1992) Microvessel quantitation and prognosis in invasive breast carcinoma. Hum Pathol 23: 755–761. 1377162 10.1016/0046-8177(92)90344-3

[bib6] de Wilt JH, ten Hagen TL, de Boeck G, van Tiel ST, de Bruijn EA, Eggermont AM (2000) Tumour necrosis factor alpha increases melphalan concentration in tumour tissue after isolated limb perfusion. Br J Cancer 82: 1000–1003. 10737379 10.1054/bjoc.1999.1032 PMC2374420

[bib7] Eggermont AM, Schraffordt KH, Klausner JM, Kroon BB, Schlag PM, Lienard D, van Geel AN, Hoekstra HJ, Meller I, Nieweg OE, Kettelhack C, Ben-Ari G, Pector JC, Lejeune FJ (1996a) Isolated limb perfusion with tumour necrosis factor and melphalan for limb salvage in 186 patients with locally advanced soft tissue extremity sarcomas. The cumulative multicenter European experience. Ann Surg 224: 756–764. 8968230 10.1097/00000658-199612000-00011 PMC1235474

[bib8] Eggermont AM, Schraffordt KH, Lienard D, Kroon BB, van Geel AN, Hoekstra HJ, Lejeune FJ (1996b) Isolated limb perfusion with high-dose tumour necrosis factor-alpha in combination with interferon-gamma and melphalan for nonresectable extremity soft tissue sarcomas: a multicenter trial. J Clin Oncol 14: 2653–2665. 8874324 10.1200/JCO.1996.14.10.2653

[bib9] Hoekstra HJ, Schraffordt KH, Molenaar WM, Oldhoff J (1987) Results of isolated regional perfusion in the treatment of malignant soft tissue tumours of the extremities. Cancer 60: 1703–1707. 2820562 10.1002/1097-0142(19871015)60:8<1703::aid-cncr2820600802>3.0.co;2-j

[bib10] Lejeune FJ, Lienard D, el Douaihy M, Seyedi JV, Ewalenko P (1989) Results of 206 isolated limb perfusions for malignant melanoma. Eur J Surg Oncol 15: 510–519. 2599121

[bib11] Lienard D, Ewalenko P, Delmotte JJ, Renard N, Lejeune FJ (1992) High-dose recombinant tumour necrosis factor alpha in combination with interferon gamma and melphalan in isolation perfusion of the limbs for melanoma and sarcoma. J Clin Oncol 10: 52–60. 1727926 10.1200/JCO.1992.10.1.52

[bib12] Lindner P, Fjalling M, Hafstrom L, Kierulff-Nielsen H, Mattsson J, Schersten T, Rizell M, Naredi P (1999) Isolated hepatic perfusion with extracorporeal oxygenation using hyperthermia, tumour necrosis factor alpha and melphalan. Eur J Surg Oncol 25: 179–185. 10218462 10.1053/ejso.1998.0623

[bib13] McBride CM (1974) Sarcomas of the limbs. Results of adjuvant chemotherapy using isolation perfusion. Arch Surg 109: 304–308. 4858720 10.1001/archsurg.1974.01360020164032

[bib15] Nooijen PT, Manusama ER, Eggermont AM, Schalkwijk L, Stavast J, Marquet RL, de Waal RM, Ruiter DJ (1996) Synergistic effects of TNF-alpha and melphalan in an isolated limb perfusion model of rat sarcoma: a histopathological, immunohistochemical and electron microscopical study. Br J Cancer 74: 1908–1915. 8980389 10.1038/bjc.1996.652 PMC2074822

[bib16] Olieman AF, van Ginkel RJ, Hoekstra HJ, Mooyaart EL, Molenaar WM, Koops HS (1997) Angiographic response of locally advanced soft-tissue sarcoma following hyperthermic isolated limb perfusion with tumour necrosis factor. Ann Surg Oncol 4: 64–69. 8985519 10.1007/BF02316812

[bib17] Ruegg C, Yilmaz A, Bieler G, Bamat J, Chaubert P, Lejeune FJ (1998) Evidence for the involvement of endothelial cell integrin alphaVbeta3 in the disruption of the tumour vasculature induced by TNF-*α* and IFN-gamma. Nat Med 4: 408–414. 9546785 10.1038/nm0498-408

[bib18] Skehan P, Storeng R, Scudiero D, Monks A, McMahon J, Vistica D, Warren JT, Bokesch H, Kenney S, Boyd MR (1990) New colorimetric cytotoxicity assay for anticancer-drug screening. J Natl Cancer Inst 82: 1107–1112. 2359136 10.1093/jnci/82.13.1107

[bib19] ten Hagen TL, Eggermont AM, Lejeune FJ (2001) TNF-*α* is here to stay--revisited. Trends Immunol 22: 127–129. 11334026 10.1016/s1471-4906(00)01850-0

[bib20] Thompson JF, Gianoutsos MP (1992) Isolated limb perfusion for melanoma: effectiveness and toxicity of cisplatin compared with that of melphalan and other drugs. World J Surg 16: 227–233. 1561803 10.1007/BF02071525

[bib21] Tjaden UR, de Bruijn EA (1990) Chromatographic analysis of anticancer drugs. J Chromatogr 531: 235–294. 2258419 10.1016/s0378-4347(00)82286-0

[bib14] United Kingdom Co-ordinating Committee on Cancer Research (UKCCCR) (1998) Guidelines for the Welfare of Animals in Experimental Neoplasia (Second Edition). Br J Cancer, 77: 1–10. 10.1038/bjc.1998.1 PMC2151254 9459138

[bib22] Vahrmeijer AL, van Dierendonck JH, Keizer HJ, Beijnen JH, Tollenaar RA, Pijl ME, Marinelli A, Kuppen PJ, van Bockel JH, Mulder GJ, van de velde CJ (2000) Increased local cytostatic drug exposure by isolated hepatic perfusion: a phase I clinical and pharmacologic evaluation of treatment with high dose melphalan in patients with colorectal cancer confined to the liver. Br J Cancer 82: 1539–1546. 10789721 10.1054/bjoc.2000.1175 PMC2363396

[bib23] van IJken MG, van Etten B, de Wilt JH, van Tiel ST, ten Hagen TL, Eggermont AM (2000) Tumour necrosis factor-alpha augments tumour effects in isolated hepatic perfusion with melphalan in a rat sarcoma model. J Immunother 23: 449–455. 10916754 10.1097/00002371-200007000-00008

[bib24] van der Veen AH, de Wilt JH, Eggermont AM, van Tiel ST, Seynhaeve AL, ten Hagen TL (2000) TNF-alpha augments intratumoural concentrations of doxorubicin in TNF-alpha-based isolated limb perfusion in rat sarcoma models and enhances anti-tumour effects. Br J Cancer 82: 973–980. 10732774 10.1054/bjoc.1999.1027 PMC2374400

[bib25] van der Veen AH, Seynhaeve AL, Breurs J, Nooijen PT, Marquet RL, Eggermont AM (1999) *In vivo* isolated kidney perfusion with tumour necrosis factor alpha (TNF-alpha) in tumour-bearing rats. Br J Cancer 79: 433–439. 10027309 10.1038/sj.bjc.6690067 PMC2362422

